# Two-stage cap-assisted endoscopic mucosal resection and papillectomy for duodenal papillary tumors with lateral growth

**DOI:** 10.1055/a-2358-1249

**Published:** 2024-07-26

**Authors:** Nobuhiko Fukuba, Masaki Onoe, Yasuhide Kodama, Satoshi Kotani, Koutarou Shibagaki, Norihisa Ishimura, Shunji Ishihara

**Affiliations:** 1 175764 Gastroenterology and Hepatology, Shimane University Faculty of Medicine Graduate School of Medicine, Izumo, Japan; 2 175764 Endoscopy, Shimane University Faculty of Medicine Graduate School of Medicine, Izumo, Japan

Endoscopic papillectomy is indicated for the treatment of duodenal papillary adenoma 1 2 . However, en bloc resection using a snare may be challenging in cases with lateral extension of tumor. For lesions that are difficult to remove en bloc with papillectomy alone, resection of two parts of the lesion with staggered timing is effective, without requiring submucosal dissection 3 . A man in his fifties was referred to our hospital following a medical examination. Duodenoscopy revealed a flat lesion arising from the papilla and extending toward the anus ( Fig. 1 ). A diagnosis of ampullary adenoma was made. The use of a snare was considered to increase the risk of residual peripheral lesions. Consequently, a strategy was devised to remove the lesion in two parts: the anal side and the ampullary side. First, we excised the anal side of the lesion using cap-assisted endoscopic mucosal resection 4 . Then, we inserted an endoscope (GIF-Q260J; Olympus, Tokyo, Japan) with a disposable hood (D-206-05; Olympus) ( Video 1 ). Following administration of physiological saline under the lesion, a 25-mm snare was set in the claw of the hood, and the anal side of the lesion was suctioned, grasped with the snare, and excised using the ENDO CUT Q mode (VAIO2; Erbe, Tübingen, Germany). Prior to resection, several trial suctions were performed to test the grip accuracy. After 8 weeks, the ulcer had scarred, and endoscopic papillectomy was performed for the residual tumor ( Fig. 2 ). Using a TJF-Q290V endoscope (Olympus, Tokyo, Japan) in the ENDO CUT Q mode, the residual tumor was excised by applying a snare from the oral side to the anal side. The treatment was completed by placing plastic stents in the bile and pancreatic ducts and applying clip sutures. The histopathological diagnosis was well-differentiated adenocarcinoma localized to the mucosa. Reconstruction of the lesion removed using cap-assisted endoscopic mucosal resection and endoscopic papillectomy revealed no residual tumor ( Fig. 3 ). No adverse events of the procedures were observed. At 1 year, endoscopy revealed no tumor recurrence.

**Fig. 1:**
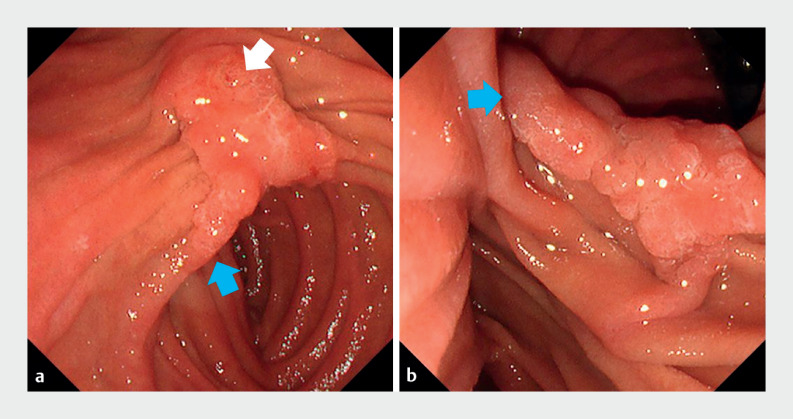
**a, b** Duodenoscopy showing the ampullary side ( **a** ) and anal side ( **b** ) of lesion with lateral extension (blue arrow). The orifice is indicated by the white arrow.

The ampullary lesion was resected in two parts. First, the anal side of the lesion was excised using cap-assisted endoscopic mucosal resection (EMR-C). After 8 weeks, endoscopic papillectomy (EP) was performed to remove the residual tumor.Video 1

**Fig. 2:**
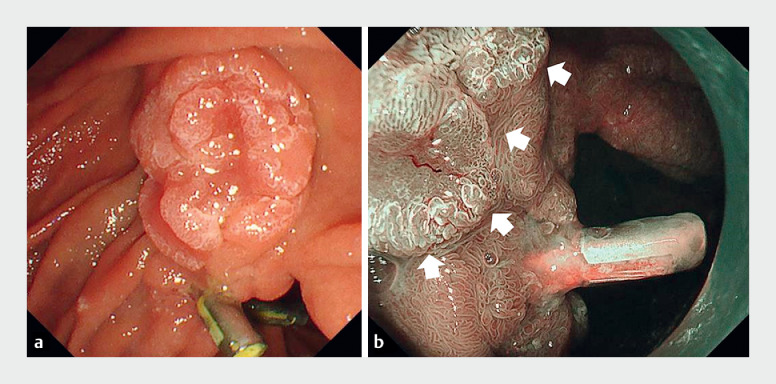
**a** Residual lesions observed using a side scope. **b** Margin of the anal side of the residual lesion (white arrow) and previously placed clip.

**Fig. 3:**
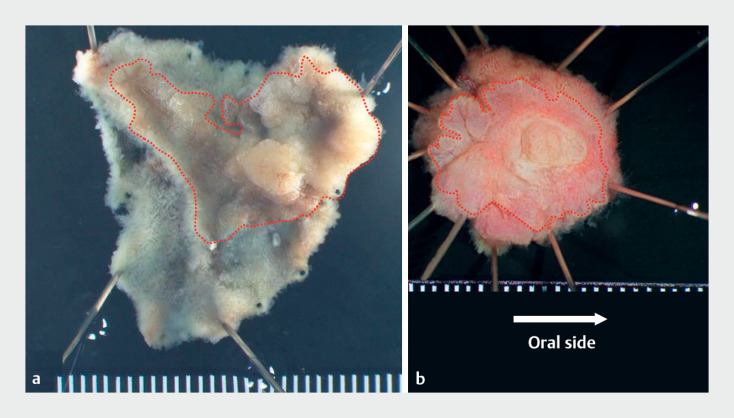
**a, b** Loupe images of specimens resected during ( **a** ) cap-assisted endoscopic mucosal resection (22 × 16 mm) and ( **b** ) endoscopic papillectomy (14 × 11 mm). The red dashed lines indicate the margin of the lesion.

Endoscopy_UCTN_Code_TTT_1AR_2AD

## Notes

Endoscopy E-Videos https://eref.thieme.de/e-videos
*E-Videos* is an open access online section of the journal *Endoscopy* , reporting on interesting cases and new techniques in gastroenterological endoscopy. All papers include a high-quality video and are published with a Creative Commons CC-BY license. Endoscopy E-Videos qualify for HINARI discounts and waivers and eligibility is automatically checked during the submission process. We grant 100% waivers to articles whose corresponding authors are based in Group A countries and 50% waivers to those who are based in Group B countries as classified by Research4Life (see: https://www.research4life.org/access/eligibility/ ). This section has its own submission website at https://mc.manuscriptcentral.com/e-videos .
